# Adult Presentation of Ectopic Vas Deferens with Dysplastic Kidney

**DOI:** 10.1089/cren.2015.0033

**Published:** 2016-01-01

**Authors:** Yusuf Saifee, Pranjal Modi

**Affiliations:** Department of Urology and Renal Transplantation, Smt. Gulabben Rasiklal Doshi and Smt. Kamlaben Mafatlal Mehta Institute of Kidney Diseases and Research Centre and Dr. H. L. Trivedi Institute of Transplantation Sciences, Ahmedabad, India.

## Abstract

A 24-year-old male presented with voiding lower urinary tract symptoms. On evaluation, the patient was found to have midbulbar urethral stricture and right dysplastic pelvic kidney with right vesicoureteral reflux. A micturating cystourethrogram (MCUG) shows opacification of the right vas deferens along the entire course till the testis. The patient underwent end-to-end urethroplasty. But soon the patient presented with urinary tract infection (UTI) and epididymorchitis in the follow-up period. The patient was explored laparoscopically to remove dysplastic kidney and ectopic vas deferens. Laparoscopically, the testicular end of the left vas deferens entering the deep inguinal ring was clipped and cut. Also the dysplastic kidney and ureter were removed till the vesicoureteral junction. At 1 year of follow-up, the patient is voiding well with no episodes of UTI.

## Introduction

Ectopic insertion of the vas deferens into the ureter is a rare phenomenon. Here we report a case ectopic vas deferens with dysplastic kidney presenting in an adult. The patient was managed by laparoscopic nephrourterectomy and vasectomy.

## Case Report

A 24-year-old male presented with voiding lower urinary tract symptoms for the past 1 year along with history of two episodes of urinary tract infection (UTI). There were no associated gastrointestinal or systemic symptoms. On clinical examination, the bladder was palpable and right epididymis was tender. Rest of the examination was unremarkable.

**FIG. 1.**
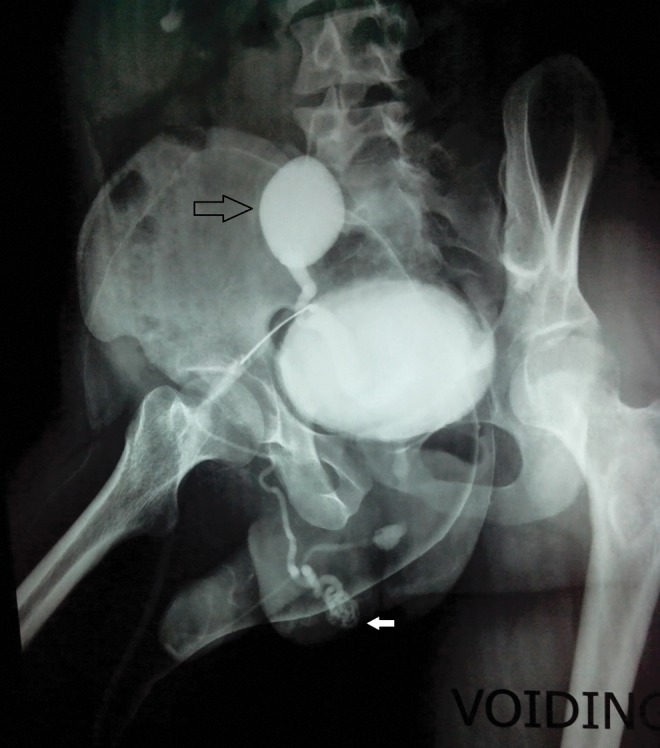
MCUG showing right vesicoureteral reflux in right dysplastic pelvic kidney (*black arrow*) and opacification of right vas deferens along the entire course till testis (*white arrow*).

**FIG. 2.**
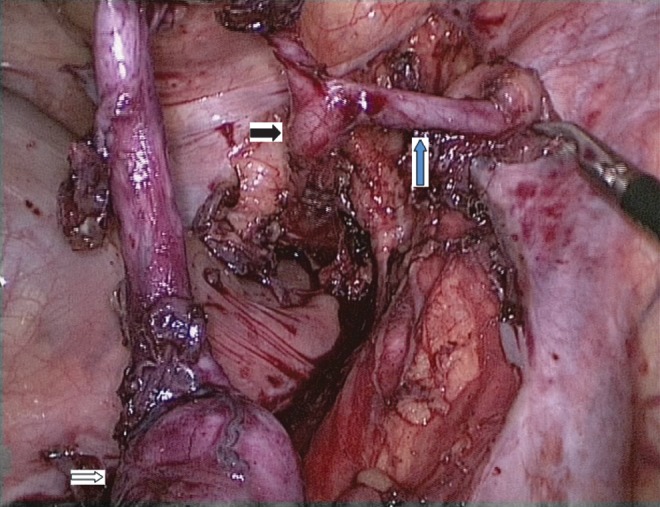
Image showing right pelvic dysplastic kidney (*white arrow*) along with ectopic vas deferens (*blue arrow*) opening in right lower ureter (*black arrow*).

Evaluation showed the right kidney was replaced by a 3 × 2 cm cyst in the right iliac fossa and a normal left kidney. Routine blood investigation was normal. Per urethral catheterization was attempted but it was not effective. Hence, suprapubic cystostomy was done. On subsequent evaluation with retrograde urethrogram and micturating cystogram (Fig. 1), the patient was found to have short midbulbar urethral stricture with right vesicoureteral reflux. Also, opacification of the right vas deferens along the entire course till testis was seen on MCUG. Thus, a diagnosis of urethral stricture with dysplastic kidney with possibility of ectopic insertion of vas deferens in ureter was made. The patient underwent end-to-end urethroplasty. Urethral catheter was removed after 3 weeks, after which the patient voided well. But soon, the patient presented with UTI and epididymorchitis in the follow-up period. The patient was explored laparoscopically to remove dysplastic kidney and ectopic vas deferens. Laparoscopically, the testicular end of the left vas deferens entering the deep inguinal ring was clipped and cut (Fig. 2). Also, the dysplastic kidney and ureter were removed till the vesicoureteral junction. At 1 year of follow-up, the patient is voiding well with no episodes of UTI.

## Discussion

Ectopic insertion of the vas deferens into the ureter is a rare phenomenon. In the developing embryo, the ureter arises as a bud from the distal Wolffian duct and grows in a cephalic direction to invaginate the portion of the nephrogenic mass that will eventually become the kidney. Because the ureter arises as a bud from the Wolffian duct, a common channel connects both structures to the urogenital sinus. With growth of the sinus, this common channel is assimilated, with the result that the ureter and future vas deferens enter separately into the primitive bladder. The part of the primitive bladder that receives the vas deferens is the precursor of the posterior urethra and carries the orifice of the vas deferens to its final position at the verumontanum.^1–4^

Failure of obliteration of the portion of the Wolffian duct that receives both the ureter and vas deferens can result in ectopy of the vas deferens into the ureter (or of the ureter into the genital tract as has also been observed). Alternatively, the vas deferens may enter ectopically into the bladder when it does not complete the final migration to the posterior urethra.^1–4^

Because these developmental events are so closely related, it is not surprising that the imperforate anus and anomalies of renal rotation and ascent are associated with congenital abnormalities of the vas deferens.^2^ Renal dysplasia is found in most of the cases of vas deferens ectopia, presumably caused by an abnormal ureteral bud.^3^

Adult presentation and association are still rarer.^4^ When associated with hydroureter because of vesicoureteral reflux or obstruction, the retrograde passage of urine into the vas deferens can cause epididymitis and even scrotal abscesses. Management of patients with ectopic vas deferens aims to prevent UTIs because of vesicovasal or vesicoureterovasal reflux causing epididymitis and scrotal abscess, or vesicoureteral reflux leading to pyelonephritis.

## Abbreviations Used

MCUG: micturating cystourethrogram
UTI: urinary tract infection
